# Hepatitis B seromarkers, hepatitis C antibody, and risk behaviors in married couples, a bordered province of western Thailand: Hepatitis B seromarkers, hepatitis C antibody, and risk behaviors

**Published:** 2011-04-01

**Authors:** Pipat Luksamijarulkul, Pittaya Piroonamornpun, Somporn Kantharadussadee Triamchaisri

**Affiliations:** 1 Department of Microbiology, Faculty of Public Health, Mahidol University, Bangkok, Thailand; 2 Tropical Medicine Hospital, Faculty of Tropical Medicine, Mahidol University, Bangkok, Thailand; 3 Department of Public Health Nursing, Faculty of Public Health, Mahidol University, Bangkok, Thailand

**Keywords:** HCV antibodies, Risk behaviors, Sex, Spouses

## Abstract

**Background:**

Married couples constitute a target group for reducing the risk of infections with hepatitis B virus (HBV) and hepatitis C virus (HCV).

**Objectives:**

This study attempted to assess HBV seromarkers, anti-HCV-positive rates, and risk behaviors among married couples in a bordered province of western Thailand.

**Materials and Methods:**

A cross-sectional study of 114 married couples aged 15-44 years was performed. Approximately 25-30 married couples were randomly selected from 4 districts in a province of western Thailand. All study participants who participated voluntarily were interviewed using structured questionnaires. Their blood specimens were collected to screen for HBV seromarkers (HBsAg, anti-HBs, and anti-HBc) and anti-HCV.

**Results:**

Approximately 21.1% of husbands and 2.6% of wives had a history of extramarital sex without using a condom; 18.4% of husbands and 4.4% of wives had tattoos; and 18.4% and 3.5%, respectively, consumed alcohol regularly. Additionally, 4.4% of husbands and 2.6% of wives had a history of sexual contact before marriage. In the serological study, 10.5% of husbands and 5.3% of wives were HBsAg-positive, and 1.8% of husbands and 0.9% of wives were anti-HCV-positive. Among HBsAg-positive subjects, 15/18 had spouses who were positive for any HBV marker, and 1 had a spouse who was HBsAg- and anti-HBc positive. Three participants were positive for anti-HCV (2 males and 1 female). One anti-HCV-positive male had a history of regular alcohol consumption and extramarital sex without a condom, and another had a history of intravenous drug use. The anti-HCV-positive female had a history of sexual contact before marriage.

**Conclusions:**

This study found high percentages of risk behaviors and HBsAg positivity among married couples in a bordered province of western Thailand, especially in husbands. These findings support the evidence of HCV transmission via sexual contact and intravenous drug use.

## Background

Sexually transmitted and bloodborne infections, especially hepatitis B virus (HBV) and hepatitis C virus (HCV), are significant public health problems in many countries, including Thailand [1][2][3][4][5][6]. There are more than 350 million HBsAg carriers worldwide and 170 million HCV-infected individuals [1][2][3][4].

The complications from HCV and HBV infections are serious. Approximately 10% to 40% will develop chronic hepatitis and experience gradual progression to liver cirrhosis and hepatocellular carcinoma (HCC) [2][3][7][8]. If HCV patients are also infected with HBV or HIV, they will develop HCC and liver cirrhosis more quickly than those who are infected with HCV alone. Additionally, HCV infection may contribute to faster progression of HIV infection [9][10][11][12][13]. The incidence of HCC and cirrhosis is nearly 6.5 to 11 times higher among HIV-coinfected patients than those without HIV coinfection [10]. HBV is transmitted parenterally and by sexual contact, whereas the principal route of HCV transmission is parenteral [2][3][4][14][15][16].

In Thailand, drug users (IDUs) and female sex workers (FSWs) are the groups at highest risk of HBV and HCV infection [14][15][17]. The spouses of IDUs and clients of FSWs have an higher chance of acquiring HBV and/or HCV if they do not use condoms at each sexual encounter. If infected, these groups can transmit the infections to the general population.

Previous studies have demonstrated sexual transmission of HBV and HCV among spouses [16][18][19]. Such infections can affect their neonates and siblings from intrafamilial transmission [20][21][22]. Married couples constitute a target group for reducing the risk of these infections. Kanchanaburi province is a bordered province in western Thailand. Risk behaviors towards sexually transmitted and blood-borne infections probably tended increase due to the high migration rate of unskilled workers from a bordered country.

## Objectives

A study of HBV seromarkers, antibodies to HCV, and risk behaviors among married couples in this province is valuable for the epidemiological surveillance and development of a special intervention program for this target group.

## Materials and Methods

### Study design and study participants

We performed a cross-sectional study between October 2004 and June 2005 of 114 married couples aged 15-44 years. Approximately 25-30 married couples, who participated voluntarily, were randomly selected from 4 districts in Kanchanaburi province, a bordered province in western Thailand. All participants, who had no history of HBV vaccination, were interviewed using structured questionnaires. Information on their socio-demographic characteristics and risk behaviors toward HBV and HCV infections was included. Blood specimens were collected to screen for HBV seromarkers (HBsAg, anti-HBs, and anti-HBc) and anti-HCV. Before the interviews and blood screening, the participants received the study information, after which they filled out informed consent forms. This study protocol was approved by the Ethics Committee of Mahidol University.

### Methods for screening blood

Blood specimens were screened for HBV seromarkers and anti-HCV using immunochromatography and immunocomb ELISA kits (ABBOTT EIA and Pacific Biotech and Organics Immunocomb II, Bangkok, Thailand) with 96% to 100% sensitivity and specificity for HBV seromarkers compared with EIA and more than 99% sensitivity and specificity for anti-HCV. For samples to have been considered HBsAg- or anti-HCV-positive, the immunochromatography and EIAmust have had to be positive.

### Data analysis

Data from interviews and blood screening were analyzed using SPSS for Windows, version 7.5, and expressed using descriptive statistics, including percentage, mean, and standard deviation. To analyze the homogeneity of the distribution of variables between 2 groups, we used the chi-square test. A critical level of p = 0.05 was considered to indicate statistical significance.

## Results

### Sociodemographic characteristics in the study couples

Of 114 married couples, 40.4% of husbands and 12.3% of wives were aged above 30 years. The mean age ± standard deviation was 28.2 ± 6.2 years for husbands and 22.1 ± 5.9 years for wives. Approximately 26.3% of husbands and 18.4% of wives had completed secondary education, and 3.5% and 1.8%, respectively, completed vocational education and undergraduate study. Most of participants had low incomes (< 10,000 baht/month). The distributions by age, occupation, and income differed significantly between husbands and wives (p < 0.001). The details are presented in Table 1.

**Table 1:** Sociodemographic characteristics of study couples (No. = 114 couples)

**Sociodemographic Characteristics**	**Husbands [No. (%)]**	**Wives [No. (%)]**	**p-value x(2)-test**
**Age **(years)			
**≤ 20**	2 (1.75)	19 (16.7)	< 0.001 a
**21– 30**	66 (57.9)	81 (71.1)	
**≥ 31**	46 (40.4)	14 (12.3)	
**Mean ± SD of Age**	28.2 ± 6.2	22.1±5.9	
**Education**			
**Primary level**	39 (34.2)	54 (47.4)	0.109
**Secondary level**	45 (39.5)	39 (34.2)	
**Vocational/undergraduate**	30 (26.3)	21 (18.4)	
**Occupation**			
**Government officer**	4 (3.5)	2 (1.8)	< 0.001 a
**Private business**	21 (18.4)	18 (15.8)	
**Laborer**	48 (42.1)	40 (35.2)	
**Agricultures**	39 (34.2)	26 (22.8)	
**Housewives/Unemployed**	2 (1.8)	28 (24.6)	
**Income (Baht/mon)**			
**≤ 5000**	33 (28.9)	70 (61.4)	< 0.001 a
**5001-10000**	56 (49.1)	27 (23.6)	
**≥ 10001**	20 (17.5)	17 (14.9)	

^a^ Statistical significance at α = 0.05

### Risk behavior toward HBV and HCV infection

Among the study couples, 21.1% of husbands and 2.6% of wives had a history of extramarital sex without a condom. Approximately 18.4% of husbands and 4.4% of wives had tattoos, and 18.4% and 3.5%, respectively, had a history of regular alcohol consumption. Further, 4.4% of husbands and 2.6% of wives had a history of sexual contact before marriage. Study husbands had a significantly higher percentage of risk behaviors than studied wives (p = 0.018). The details are shown in Table 2.

**Table 2:** Risk behaviors toward HBV and HCV infection among study couples

**Risk Behaviors**	**Husbands **(No.=114) No. (%)	**Wives **(No.=114) No. %
**History of receiving blood and/or hemodialysis**	6 (5.3)	8 (7.0)
**History of jaundice**	12 (10.5)	8 (7.0)
**History of tattooing**	21 (18.4)	5 (4.4)
**History of intravenous drug use**	1 (0.9)	0 (0.0)
**History of regular alcohol consumption**	21 (18.4)	4 (3.5)
**History of STDs in the previous year**	9 (7.9)	1 (0.9)
**History of extramarital sexual relations without a condom in the previous year**	24 (21.1)	3 (2.6)
**History of sexual contact before marriage**	5 (4.4)	3 (2.6)
**p-value from x(2)-test**	0.018 a

^a^ Statistical significance at α = 0.05

### Prevalence of HBV seromarkers and anti-HCV

Of the 114 studied couples, 53.5% of husbands and 46.5% of wives were positive for HBV markers. Approximately 10.5% of husbands and 5.3% of wives were HBsAg-positive, and 1.8% of husbands and 0.9% of wives were anti-HCV-positive. Study husbands had relatively higher overall HBV markers and HBsAg-positive and anti-HCV rates than study wives (p = 0.354), Table 3. Among 18 HBsAg-positive subjects, 15 had spouses who were positive for any HBV marker, and 1 had an HBsAg- and anti-HBc-positive spouse (Table 4). In addition, 3 participants were positive for anti-HCV (2 males and 1 female). Of the 2 anti-HCV positive males, 1 had consumed alcohol regularly and had a history of extramarital sex without a condom, and the other had a history of intravenous drug use. The anti-HCV positive female had a history of sexual contact before marriage and no history of other risk behaviors (Table 3).

**Table 3:** Prevalence of HBV seromarkers and anti-HCV among study couples

**Variables**	**Numberof tested**	**HBV Seromarker positive**				**Anti-HCV positive**
		**HBsAg , Anti-HBc**	**Anti-HBs, Anti-HBc**	**Anti-HBc only**	**Any HBV markers**	
**Married status**						
**Husband**	114	12 (10.5)	31 (27.2)	18 (15.8)	61 (53.5) a	2 (1.8)^b^
**Wife**	114	6 (5.3)	27 (23.7)	20 (17.5)	53 (46.5) a	1 (0.9) c
**Total**	228	18 (7.9)	58 (25.4)	38 (16.7)	114 (50.0)	3 (1.3)

^a^ p-value from x(2)-test = 0.354

^b^ One had regular alcohol consumption and a history of extramarital sex without a condom, and the other had a history of intravenous drug use.

^c^ Had a history of sexual contact before marriage and no history of other risk behaviors

**Table 4:** Characteristics of 18 HBsAg-positive subjects and HBV seromarker status in their spouses

**Serial Number**	**Titer of HBsAgin subjects**	**Gender**	**Duration of marriage **(mon)	**HBV Seromarkers of married couple**
**1**	< 1:80	Female	32	Negative
**2**	< 1:80	Female	59	Anti-HBs + Anti-HBc
**3**	1:80	Male	30	Anti-HBs + Anti-HBc
**4**	1:80	Female	51	Anti-HBs + Anti-HBc
**5**	1:80	Female	36	Negative
**6**	1:80	Male	37	Negative
**7**	1:320	Male	12	Anti-HBc only
**8**	1:320	Male	18	Anti-HBs + Anti-HBc
**9**	1:640	Male	41	Anti-HBs + Anti-HBc
**10**	1:640	Male	52	Anti-HBs + Anti-HBc
**11**	1:640	Male	52	Anti-HBs + Anti-HBc
**12**	1:1280	Female	60	HBsAg + Anti-HBc
**13**	1:1280	Male	24	Anti-HBc only
**14**	1:2560	Female	48	Anti-HBc only
**15**	1:2560	Male	59	Anti-HBc only
**16**	1:5120	Male	48	Anti-HBs + Anti-HBc
**17 a**	1:5120	Male	60	HBsAg + Anti-HBc
**18**	1:10240	Male	54	Anti-HBc only

^a^ Both husband and wife were positive for HBsAg and anti-HBc

## Discussion

Both sexual and parenteral routes are the predominant modes of HBV transmission. In adults, this transmission is primariily person-to-person, via sexual contact. The spouses of HBV carriers can acquire the virus from them and spread it to others [3][4][5][19]. In contrast, the major route of HCV transmission is parenteral [2][14][15][18]. This study identified a wife who was positive for anti-HCV and had a history of sexual contact before marriage and no other risk behaviorsand 1 of 2 positive anti-HCV husbands who had a history of extramarital sex without a condom; the other had a history of intravenous drug use. These findings demonstra the sexual transmission of HCV. Both HBV and HCV infections can affect neonates and siblings from intrafamilial transmission [20][21][22].

This study of married couples in a bordered province of western Thailand observed a high percentage of important risk behaviors, such as a history of extramarital sex without a condom (21.1% of husbands and 2.6% of wives), regular alcohol consumption (18.4% of husbands and 3.5% of wives), tattoos (18.4% of husbands and 4.4% of wives), and a history of sexual contact before marriage (4.4% of husbands and 2.6% of wives). Tattoos, extramarital sexual relations, and sexual contact are known risk factors for HBV infection [3][16][19][23]. Alcohol consumption is an indirect risk behavior for HBV infection, because after consumption, the subject is more prone to have extramarital sexual relations without the use of a condom [23]. Intravenous drug use is an important risk factor of HCV and HBV infection [1][2][4][15][16][17][19][20].

Males had a relatively higher percentage of risk behaviors than female, consistent with several studies [3][23][24][25]. Additionally, the HBsAg-positive rate in study husbands (10.5%) was relatively higher than in other studies in the Thai population (4.3-6.1%), whereas anti-HCV positivity was similar to other reports [4][24][25]. However, false positives for anti-HCV can occur in a low-risk group; thus, the anti-HCV results in this study were confirmed by 2 screens-immunochromatography and EIA. To reduce these risk behaviors, integrated preventive measures, including premarital counseling, life skill education, and a 100% condom use policy, should be implemented. Additionally, HBV vaccination in this target group is urged to prevent HBV infection.
